# When Do those “Risk-Taking Adolescents” Take Risks? The Combined Effects of Risk Encouragement by Peers, Mild-to-Borderline Intellectual Disability and Sex

**DOI:** 10.1007/s10802-020-00617-8

**Published:** 2020-01-17

**Authors:** Eline Wagemaker, Hilde M. Huizenga, Tycho J. Dekkers, Annematt L. Collot d’Escury-Koenigs, Elske Salemink, Anika Bexkens

**Affiliations:** 1 Department of Psychology, University of Amsterdam, Nieuwe Achtergracht 129b, 1018 WS Amsterdam, The Netherlands; 2 Research Priority Area Yield, University of Amsterdam, Amsterdam, The Netherlands; 3 Amsterdam Brain and Cognition, University of Amsterdam, Amsterdam, The Netherlands; 4 Department of Forensic Youth Psychiatry and Behavioral Disorders, De Bascule, Academic Center of Child- and Adolescent Psychiatry, Amsterdam, The Netherlands; 5 Mind at Work, Almere, The Netherlands; 6 Department of Clinical Psychology, Utrecht University, Utrecht, The Netherlands; 7 Department of Psychology, Developmental and Educational Psychology, Leiden University, Leiden, The Netherlands; 8 GGZ Delfland, Delft, The Netherlands

**Keywords:** Adolescence, Risk taking, Peer influence, Intellectual disability, Balloon Analogue Risk Task

## Abstract

Adolescents with mild to borderline intellectual disability (MBID) show more daily life risk taking than typically developing adolescents. To obtain insight in when these “risk-taking adolescents” especially take risks, we investigated main and interaction effects of (a) MBID, (b) sex, and (c) type of peer influence on risk taking. The Balloon Analogue Risk Task (BART) was used as a proxy of real-life risk taking. 356 adolescents (12–19 years, 51.7% MBID, 63.4% boys) were randomly assigned to one of three BART peer-influence conditions: solo (no peers), positive risk encouragement (e.g., *‘You are cool if you continue’*) or negative risk encouragement (e.g., *‘You are a softy if you do not continue’*). The main finding was that boys with MBID took more risks than typically developing boys in the negative risk encouragement condition. Boys with MBID also took more risks in the negative risk encouragement condition compared to the solo condition, whereas typically developing boys did not. There were no such effects for girls. Surprisingly, boys with MBID took less risks in the solo condition than typically developing boys. We conclude that boys with MBID especially show high risk taking when peers belittle or threat with exclusion from the peer group. Prevention and intervention programs should specifically target boys with MBID to teach them to resist negative risk encouragement by peers.

Risk-taking behavior (e.g., substance use, reckless driving, sexual risk taking) is the leading cause of death in adolescents (Dahl 2004; Institute of Medicine 2011) and results in high societal costs (Groot et al. 2007). The dual systems model or imbalance model provides the dominant explanation for high risk taking in adolescence by proposing an imbalance between the fast developing social-emotional systems and the more gradual developing cognitive control systems in the adolescent brain (Steinberg 2010). However, not all adolescents show high risk taking, many individual differences exist. In 2015, Bjork and Pardini published a paper centered on the question ‘*Who are those “risk-taking adolescents”?*’. Based on their research and earlier findings, high risk taking in adolescence has been related to sensation seeking, impulsivity, low cognitive control, and low educational levels (Bjork and Pardini 2015; Harakeh et al. 2012; Jessor 1992). These characteristics are often overly represented in adolescents with mild to borderline intellectual disability (MBID; e.g., Bexkens et al., 2014). MBID is defined by an IQ between 50 and 85 and problems in social adaptability (De Beer 2016). The prevalence of MBID is about 10% (Simonoff et al. 2006). In daily life, adolescents with MBID indeed show more risk taking than typically developing peers, and they are over-represented in the criminal justice system (Holland et al. 2002; Kaal 2016; Van Duijvenbode and Van der Nagel 2019). However, it is unclear whether all adolescents with MBID are risk takers and whether the risk-taking context matters. Therefore, the current study examines the impact of sex and peer influences on risk taking in adolescents with and without MBID.

Adolescence is known as a period of increased sensitivity to peer influence (Crone and Dahl 2012; Steinberg and Morris 2001; Van Hoorn et al. 2017). Indeed, the peer context is related to adolescent risk taking in daily life, as demonstrated by the doubled risk of fatal injuries during driving accompanied by peers (Chen et al. 2000). Several experimental studies also demonstrate that the (perceived) presence of peers increases risk taking (De Boer and Harakeh 2017; Gardner and Steinberg 2005; Maclean et al. 2014; Van Hoorn et al. 2017; Weigard et al. 2014). However, adolescents vary in their susceptibility to peer influence on risk taking. These individual differences can be related to resistance skills, social acceptance by friends or peer status (Allen et al. 2012; Prinstein et al. 2011; Urberg et al. 2003). More knowledge about individual factors could help to identify specific contexts in which at-risk groups are likely to show risky behavior. With this knowledge, prevention or intervention programs could be fine-tuned. The current study focuses on three potential factors that may affect susceptibility to peer influence in a risk-taking context. First, low intellectual functioning (i.e., MBID) could increase the susceptibility to peer influence. Second, boys and girls could differ in their susceptibility to peer influence. Third, the type of risk encouragement by peers may differentially relate to risk taking. The literature for each of these factors is reviewed below.

## Susceptibility to Peer Influence in Adolescents with MBID

Few studies have focused on susceptibility to peer influence in adolescents with MBID. Professionals working with this population, however, often report questions on how to deal with risk taking related to increased susceptibility to peer influence. Indeed, intellectual disability is characterized by lower risk-awareness (Greenspan et al. 2011), and vignette studies suggest that adolescents with MBID especially struggle to make safe decisions under peer influence (Khemka et al. 2009). Also, adolescents with MBID report lower resistance to peer influence than typically developing adolescents (Dekkers et al. 2017). To our knowledge, there is only one experimental study that compared the susceptibility to peer influence of adolescents with MBID and typically developing adolescents (Bexkens et al. 2018). In this study, boys with MBID and typically developing boys performed a risk-taking task either with or without three pictures of same-sex virtual peers that gave risk encouraging feedback. Both groups demonstrated more risk taking in the version with the virtual peers than in the version without the peers. On top of that, adolescents with MBID took more risks than typically developing adolescents, but only in the condition with the virtual peers. This study provides initial evidence that adolescents with MBID may be more susceptible to peer influence than typically developing adolescents. From the perspective of the imbalance model, increased susceptibility to peer influence in adolescents with MBID could be explained by a larger imbalance between social-emotional and cognitive control systems. Although this claim has not been investigated at a neurobiological level, there is ample evidence that adolescents with MBID have inhibition deficits as compared to typically developing adolescents (see Bexkens et al. 2014 for a meta-analysis; Schuiringa et al. 2017), and inhibition is an important component of cognitive control (Ridderinkhof et al. 2004). These inhibitory deficits may suggest a larger imbalance between the social-emotional and the cognitive control system in adolescents with MBID, which may increase their susceptibility to peer influence (Albert et al. 2013). Therefore, the first goal of this study is to confirm that adolescents with MBID are more susceptible to peer influence than adolescents without MBID.

## Sex Differences in Susceptibility to Peer Influence

Previous research about sex differences in susceptibility to peer influence showed mixed results. Several experimental and self-report studies found that boys were more susceptible to peer influence than girls (De Boer et al. 2017; Sumter et al. 2009; Widman et al. 2016), and this pattern was similar for adolescents with MBID (Dekkers et al. 2017). However, other experimental studies demonstrated that girls are more susceptible to peer influence than boys (Shepherd et al. 2011) or did not find any differences between boys and girls (Gardner and Steinberg 2005). Similarly, recent neurobiological studies propose a general pubertal surge in testosterone in both boys and girls, which increases the motivation to be admired by others, and may therefore increase risk taking under peer influence in both boys and girls (Braams et al. 2015; Crone and Dahl 2012). Based on these studies, it remains unclear whether sex differences are relevant in susceptibility to peer influence. Therefore, the second goal of this study is to explore sex differences in susceptibility to peer influence in adolescents with and without MBID.

## The Effect of the Type of Risk Encouragement by Peers

Peers can actively encourage risk taking in a variety of ways. In Bexkens et al. (2018), peers encouraged risk taking in a mixed way: some feedback had a positive tone (e.g., giving compliments), whereas other feedback had a more negative tone (e.g., belittling). Consequently, this study did not allow to test differential effects of positive and negative risk encouragements on risk taking. Therefore, we tested whether these different encouragements have differential effects by assigning adolescents to either a positive or a negative risk encouragement condition. In the positive condition peers give compliments or talk about inclusion in the peer group, while in the negative condition peers belittle or threat with exclusion from the peer group.

Three lines of evidence suggest that negative risk encouragement by peers, especially in the form of threatening with social exclusion, can increase risk taking in adolescents. First, because forming peer relations is important in adolescence, the perception of being socially rejected can lead to a process called reputation management (i.e., displaying risk taking to gain status or avoid rejection by peers; Blakemore 2018; Brechwald and Prinstein 2011; Chen et al. 2015). Second, perceived peer rejection causes stress in adolescents (Gunther Moor et al. 2014), which has a negative impact on inhibition (De Houwer and Tibboel 2010) and may therefore foster risk taking (Bjork and Pardini 2015). This is also in line with the need-to-belong theory predicting that social exclusion leads to decreased self-regulation (Baumeister et al. 2005; DeWall et al. 2008; Stenseng et al. 2015). Finally, longitudinal studies confirm that a negative interactional style between adolescents (i.e., negative laughter and coercive statements) predicts adolescents’ school misconduct and risk taking (Ellis et al. 2018)*.*

Positive risk encouragements such as laughing or giving positive feedback in response to risk-taking behavior are highly prevalent in deviant adolescents (Dishion et al. 1999). Arguably, positive risk encouragement by peers could increase risk taking through the same mechanisms as negative risk encouragement, although in a more indirect way. That is, adolescents who receive compliments from peers on risk taking may get the impression that peers will not exclude them if they take risks. Thus, to avoid exclusion, these adolescents will show risk taking in response to positive risk encouragement.

To our knowledge, there is only one experimental study in children (10 years) that compared the effects of social exclusion versus inclusion on risk taking (Nesdale and Lambert 2008). This study showed that children demonstrated more risk taking after social exclusion than after social inclusion. As this study did not explicitly focus on what peers say to encourage risk taking, the evidence on differential effects of peer encouragements is limited. Therefore, the third goal of this study is to explore the effects of negative and positive risk encouragement by adolescent peers on risk taking.

## The Current Study

To disentangle the effects of MBID, sex, and risk encouragement by peers on risk taking, we adopt a 2 (MBID: present vs. absent) by 2 (sex: boys vs. girls) by 3 (type of risk encouragement by peers: positive vs. negative vs. none) between-subjects design. To measure susceptibility to peer influence, we use a risk-taking task with a virtual peer paradigm as this enables standardization of peer influence. It has been shown that this paradigm yields similar effect sizes as paradigms with real peers (Chein et al. 2011; Festl et al. 2013; Gardner and Steinberg 2005; O’Brien et al. 2011; Reynolds et al. 2014; Weigard et al. 2014). We manipulated the type of risk encouragement during the task and propose the following hypotheses. First, we hypothesize that all adolescents show increased risk taking when exposed to peer influence compared to solo risk-taking. As previous research provides limited results about whether positive or negative peer encouragement has a stronger effect, we expect that the effect of peer condition on risk taking is present for both positive and negative risk encouragement by peers. Second, we hypothesize that adolescents with MBID show more risk taking than typically developing adolescents, especially when exposed to peer influence. Again, as previous research is insufficient to claim that positive and negative peer encouragement have differential effects, we expect a similar effect of MBID on risk taking for positive and negative peer encouragement. Third, we explore (a) the differences between boys and girls as well as interactions between sex, type of peer encouragement, and MBID and (b) the differences between positive and negative peer encouragement. Fourth, we explore the validity of the risk-taking task by relating it to scores on a self-report questionnaire measuring real-life susceptibility to peer influence (Cavalca et al. 2013).

## Methods

### Participants

382 adolescents between 12 and 19 years were recruited at secondary schools (*M*_*age*_ = 15.31, *SD* = 1.42, 63.4% boys). Assignment to the MBID and the control group was based on school type. Adolescents from special vocational education schools were assigned to the MBID group. The current study took place in the Netherlands. Special education schools for MBID and practical education schools in the Netherlands have the following admittance criteria: (1) an IQ between 60 and 85 tested no more than 2 years prior to admittance; and (2) learning delays of 50% or more in at least two of the following areas: mathematics, reading accuracy and fluency, reading comprehension, and spelling.^1^ Adolescents from Dutch regular education secondary schools with different educational levels (i.e. lower and higher vocational education and pre-university education) were assigned to the control group. To confirm that the MBID and the control group actually differed in IQ score, we administered Raven’s Standard Progressive Matrices (Raven’s SPM; Raven et al. 1988).

Informed consent was obtained from both parents (or caretakers) and adolescents. Schools sent passive consent letters to parents two weeks prior to testing. Adolescents provided their assent immediately before testing. It was explained to both parents and adolescents that they could withdraw from participation whenever they wanted and without consequences. The study was approved by the ethical review board of the University of Amsterdam and complied with relevant laws and guidelines.

## Materials

### Balloon Analogue Risk Task (BART)

**Fig. 1:**
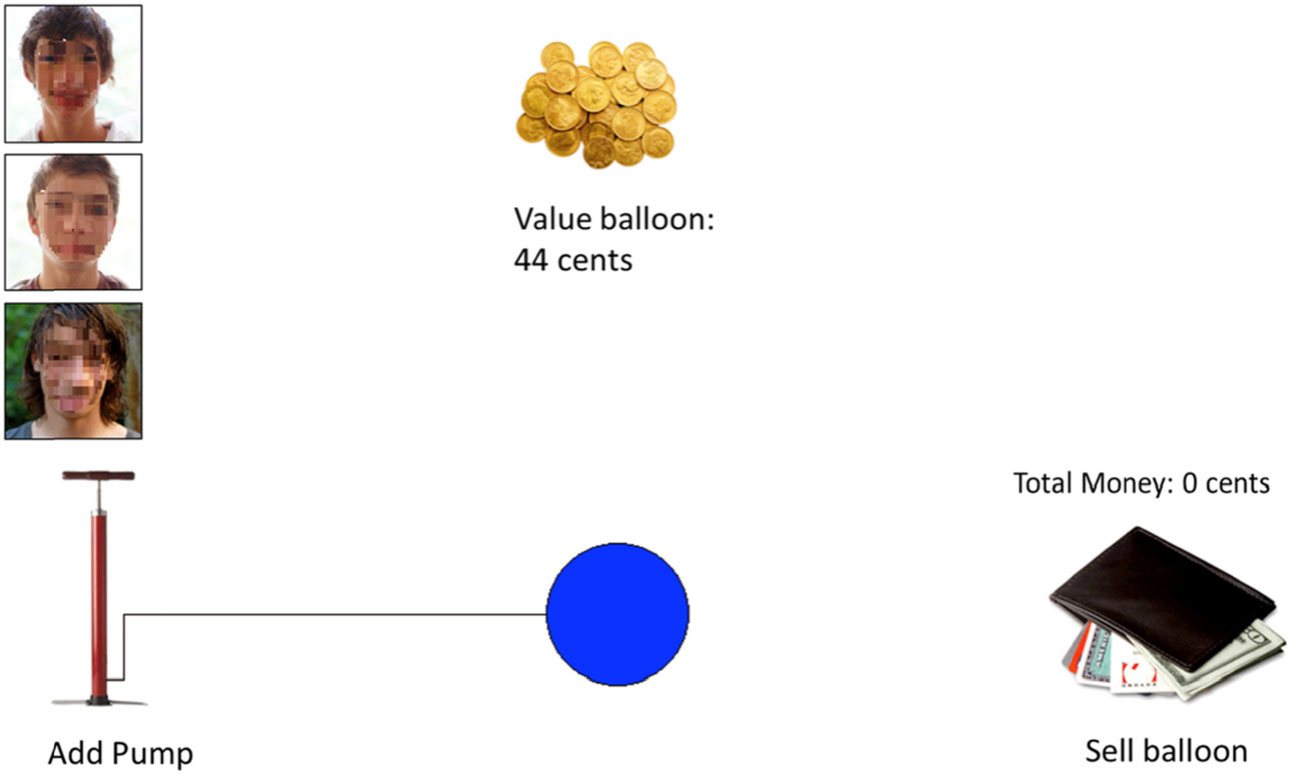
Sample trial from one of the peer influence conditions (faces are blurred to ensure anonymity)

An adaptation of the computerized BART (Lejuez et al. 2002, adapted from Bexkens et al. 2018) was used to assess risk-taking. The task was presented on a HP 550, 15.4-in. notebook. On each trial, participants were instructed that they could earn money by inflating a balloon, whilst simultaneously running the risk of popping the balloon and losing the money. Participants inflated the balloon by clicking the pump (see Fig. 1). Each click inflated the balloon a little, which was depicted visually, and was rewarded with one cent. A counter above the balloon kept track of the number of cents earned on a particular trial. Participants risked the balloon to explode at each next click. When the balloon exploded, an explosion cartoon was presented on screen together with an explosion sound. All cents earned on that trial were lost and the counter above the balloon was reset to zero. Participants were instructed that they could decide to ‘sell’ the balloon at any point of their own preference by clicking on the picture of a wallet. Money earned on that trial was then transferred to the counter above the wallet, which was accompanied by the sound of a slot machine. This money remained in possession of the participant and could be exchanged for raffle tickets at the end of the session (Lejuez et al. 2003; Lejuez et al. 2002).

Our adaptation based on Bexkens et al. (2018) differed from the original procedure (Lejuez et al. 2002) in that the balloon could not explode on the first five pumps. The probability of explosion on the sixth pump was 1/123, the probability of explosion on the seventh pump was 1/122, etc. This probability distribution was used once to generate an explosion point for every balloon. This array of explosion points was then used for all participants, so there was no inter-participant variation in the probability of exploding. The mean point of explosion was 57.6 pumps. The number of adjusted pumps (i.e., the average number of pumps on non-explosion trials) was used as dependent variable.

The BART has a test-retest correlation of *r* = 0.77 across days (White et al. 2008). Construct validity is supported by significant relations to a range of daily life risk-taking behaviors (Hunt et al. 2005; Lejuez et al. 2002; MacPherson et al. 2010; Mishra et al. 2010). The adjusted current version of the BART was used successfully in adolescents with MBID before (Bexkens et al. 2018).

### Peer Influence Manipulation

Three conditions were used: no peer influence (i.e., the solo condition), negative risk encouragement by peers, and positive risk encouragement by peers. These BART conditions were based on the solo and peer condition of Bexkens et al. (2018) but had one important difference: we split the positive and negative risk encouragements into two separate conditions. Participants were randomly assigned to one of the three BART conditions. In the solo condition, there was no peer influence. In the negative and positive risk encouragement conditions, risk encouragement by peers was added visually and auditory. Three pictures of same-sex peers were displayed during each trial of the BART and audio files with risk encouraging statements were played in each trial (see Fig. 1). When an audio file played, a speech balloon appeared next to one of the pictures indicating which peer was speaking. Participants were instructed that these peers already completed the task and would give feedback based on the participants’ performance. In the positive risk encouragement condition, the statements were positively formulated and/or contained signs of inclusion (e.g., ‘*Yes continue’* or *‘You belong to us if you continue’*). In the negative risk encouragement condition, the statements were negatively formulated and/or contained signs of exclusion (e.g., ‘*Continue softy’* or *‘If you stop you are chicken’*). A pilot study was performed on the reliability of the risk-encouraging statements before execution of the study. 41 first year psychology students rated 243 statements on a 5-point positivity-negativity scale and on an inclusion-exclusion scale. Both scales had high reliability (positivity-negativity scale *α* = 0.96, inclusion-exclusion scale *α* =0.95). The 183 statements with most reliable scores were used in the BART. The statements were played in a random order and at semi-random moments during each trial (i.e., never on one of the first 10 pumps, always separated by more than 10 pumps, and the same maximum number of statements per balloon). A supplement containing all the Dutch statements can be requested from the corresponding author.

### Exit Interview

In a random subgroup of the MBID group, an exit interview was added in which participants were questioned about their general comprehension of the BART and about the peer manipulation. The results suggested that participants sufficiently comprehended the BART-related reward, could distinct the positive and negative risk encouragement, and in general did not trust the feedback of the peers. Specific questions and results can be found in Appendix 1.

### Raven’s Standard Progressive Matrices (Raven’s SPM)

Intelligence was estimated using Raven’s SPM (Raven et al. 1988), which is a figural test using 60 items delivered in 5 sets. Each item consists of a series of figures following a certain logical pattern in which one figure is missing, as indicated by an empty square. The participant was asked which figure from 8 options should be put in the empty square. Within each set, the items became progressively more difficult. The sets in turn also became progressively more difficult. The maximum score is 60, higher scores indicate higher cognitive abilities. Raven’s SPM has high internal consistency and validity (Lynn and Irwing 2004).

### Resistance to Peer Influence Scale (RPI)

We used the RPI (Steinberg & Monahan, 2009) as a self-report measure of susceptibility to peer influence. The RPI consists of ten items about the ability to resist peer influences. On each item, participants were first asked to choose the option that best described the group of people they belonged to (i.e., more vs. less peer resistant), and then they were asked to indicate to what degree they feel they belong to this group (i.e., “Really true” or “Sort of true”). Scores on each item were aggregated in a 4-point Likert-type scale score, in which the “Really true” and “Sort of true” options of the less peer-resistant statement were coded as 1 and 2 respectively, and the “Sort of true” and “Really true” options of the more peer-resistant statement were coded as 3 and 4 respectively. A sample item was: “Some children will not break the law just because their friends say that they would BUT other children would break the law if their friends said that they would break it”. The maximum score is 40 and higher scores indicate higher resistance to peer influence. The reliability of the RPI is high (*α* > 0.70; Steinberg and Monahan 2007) and criterion validity has been demonstrated (Monahan et al. 2009a, b; Steinberg and Monahan 2007).

To make sure that participants understood the structure of the items of the RPI, the research assistants provided additional assistance. First, the research assistant read the instructions aloud. Participants were then asked to read aloud the sample item (“Some children like to do fun things with a lot of people BUT other children like to do fun things with just a few people”) and to verbally indicate how they would answer the item. This procedure was repeated, if necessary, until participants understood the structure of the items and understood what was required from them. Before completing the questionnaire, participants were instructed to ask for clarification if needed. Participants then completed the questionnaire, while the experimenter stayed in another part of the room to answer questions when needed. The RPI data of the current sample were also published in another paper that demonstrated that adolescents with MBID were able to understand the RPI (Dekkers et al. 2017).

### Procedure

Schools willing to participate sent out the passive consent letters to parents. Then, research assistants visited the adolescents that were permitted to participate during their classes. During this visit, all adolescents received general information about the test procedure. All tests were performed individually at school on laptops in a separate testing room. Adolescents provided their assent immediately before testing. First, participants completed the BART. A standardized step-by-step instruction including two example items was programmed into the task and was read aloud by the research assistant. After these instructions, participants were first asked whether they had any questions. Then, participants were told that they could receive a ticket per 100 cents gained to join a raffle and were asked to choose one of four age-appropriate potential raffle prizes worth 75 euros (portable DVD player, iPod shuffle, national entertainment gift card, or a general gift voucher of 75 euros) by clicking the corresponding picture on the screen. After this, participants completed the BART, while the experimenter stayed in another part of the room to answer questions when needed. After finishing the BART, participants received the raffle tickets. In each participating school, there was one winner of the raffle, which was also told to the participants. Participants took about 20 min to complete the task. All adolescents were able to perform on the task. Additional instruction was provided when needed during the practice trials. Second, participants received instructions on the RPI. If necessary, an example question was answered together with the research assistant. Then, participants filled in the RPI individually, which took about five minutes. Third, the Raven’s SPM was administered. In the MBID group, a random subgroup filled in the exit interview. At the end, participants received raffle tickets based on the BART score and a small chocolate bar.

### Data Analysis

MBID was used as a categorical predictor in the analyses because MBID is a well-defined distinct diagnostic category (American Psychiatric Association 2013; De Beer 2016). Knowledge about individual or contextual moderators of risk taking in adolescents with MBID is highly important, as it can guide interventions and future research for this highly prevalent and seriously impaired group (Simonoff et al. 2006). The control group was coded as 0 and the MBID group as 1. Age was added as covariate, as adolescents in the MBID group (*M* = 15.68, *SD* = 1.72) were significantly older than adolescents in the control group (*M* = 14.91, *SD* = 0.85; *t*(27.18) = 5.46, *p* < 0.001). The assumption of homogenous regression lines was met as age did not interact with MBID and all other independent variables (main effect age: *F*(7,314) = 0.88, *p* = 0.52; age × MBID: *F*(4,314) = 0.19, *p* = 0.94; age × sex: *F*(6,314) = 0.50, *p* = 0.81; age × BART condition: *F*(12,314) = 0.78, *p* = 0.68). Moreover, age had no significant linear, quadratic or cubic effect on risk taking, both when all BART conditions were analyzed combined, and separately (all *p*’s > 0.05). Outliers were detected with the Median Absolute Deviation method (MAD; Leys et al. 2013) within the MBID and sex subgroups.

To investigate the effects of MBID (present vs. absent), sex (boys vs. girls), and BART condition (solo vs. positive vs. negative) on the number of adjusted pumps in the BART, we performed a 2 × 2 × 3 factorial ANCOVA. In addition to the main effects, all interactions were included in the ANCOVA: MBID × sex, MBID × BART condition, sex × BART condition, and MBID × sex × BART condition. Whenever appropriate, we calculated partial eta squared effect sizes (denoted by *η*_*p*_2) which can be interpreted as small (*η*_*p*_^*2*^ = 0.01), medium (*η*_*p*_^*2*^ = 0.06) or large (*η*_*p*_^*2*^ = 0.14; Cohen 1988). An a priori power analysis revealed that a minimum of 158 participants was sufficient to achieve a power of 0.8 and detect main effects and two- and three-way interactions with a medium effect size (*f* = 0.25).

For all follow-up tests, we used Bonferroni corrected post-hoc tests in SPSS. By using this correction, *p* values in the SPSS output are multiplied by the number of tests performed (IBM Support 2016). SPSS also returns the mean difference between the groups of the simple comparison, which will be reported as *∆M*. To be consistent, we also applied the Bonferroni correction when testing simple effects. For example, to follow-up the interaction between MBID and BART condition by testing the effect of MBID in each of the three BART conditions, we multiplied the *p* value by three. We performed the Bonferroni correction for each follow-up test separately. All Bonferroni corrected *p*-values are denoted by *p*^*B*^*.* We calculated *η*_*p*_^*2*^ effect sizes for ANOVA’s and Cohen’s *d* effect sizes for *t-*tests (small: *d =* 0.2, medium: *d* = 0.5, large: *d* = 0.8; Cohen 1988).

With regard to the first hypothesis, we expected a significant main effect of BART condition with the positive and negative risk encouragement conditions having a higher number of adjusted pumps than the solo condition. With regard to the second hypothesis, we expected a main effect of MBID with the MBID group having a higher number of adjusted pumps than the control group in all BART conditions. Also, we expected a MBID × BART condition interaction with the MBID group especially having a higher number of adjusted pumps than the control group in the positive and negative risk encouragement conditions. Third, we explored (a) the main effect of sex and interactions including sex and (b) potential differences in risk taking between the positive and the negative risk encouragement condition, also in interaction with MBID and sex. Fourth, to explore the validity of the experimental BART peer influence conditions as a measure of susceptibility to peer influence, we related these BART scores to a real-life indicator of susceptibility to peer influence: the RPI score.^2^ That is, in each of the BART peer influence conditions (i.e. positive or negative risk encouragement condition), we calculated partial correlations between RPI score and adjusted pumps while controlling for age. We did this for the whole sample and for the MBID and control group separately. To test whether the potential relationship between RPI score and adjusted pumps differed between the MBID and the control group, we performed two follow-up ANCOVA’s in each of the BART peer influence conditions. We included the main effects of RPI and MBID, the interaction between RPI and MBID, and age as covariate. When the interaction term is significant, this proves that the relation between RPI and adjusted pumps differs between the MBID and the control group. Outliers on the RPI score were also detected with the MAD method within the MBID and sex subgroups.

## Results

### Preliminary Analyses

From the 382 recruited participants, 17 participants were excluded for the following reasons: the BART was not finished (4), some trials of the BART were missing (1), the number of adjusted pumps equalled zero because none of the balloons were sold (1), Raven’s SPM scores were missing (2), Raven’s SPM score equalled 0 (3), or RPI scores were missing (6). 13 of the 17 excluded participants belonged to the MBID group. Of the remaining 365 participants, eight participants had an outlying score on the BART and one had an outlying score on the RPI. These nine participants were equally distributed over all three predictors (4 controls / 5 MBID, 5 boys / 4 girls, 1 solo / 4 positive / 4 negative risk encouragement condition) and were also excluded, leaving a total sample size of 356 participants.

From 144 of the 184 MBID participants school file information about DSM-IV classifications was available. This revealed that 25% of the adolescents in the MBID group had a clinical diagnosis: Attention Deficit Hyperactivity Disorder (ADHD; 8), Oppositional Defiant Disorder (ODD; 8), ADHD/ODD (2), parent-child relational problem (2), parent-child relational problem + ADHD (2), parent-child relational problem + ODD (1), behavior disorder not otherwise specified (1), Autism Spectrum Disorder (1), Pervasive Developmental Disorder-Not Otherwise Specified (6), attachment disorder (2), adjustment disorder (1), dyslexia + ADHD (1), and parent-child relational problem + Post-Traumatic Stress Disorder (1). Overall, the majority of this group had comorbid externalizing disorders (ADHD, ODD), which have been related to risk taking (Dekkers et al. 2016; Pollak et al. 2019).

Chi-square tests confirmed that the MBID and the control group were not significantly different in boy/girl ratio (60.9% boys in MBID group / 66.3% boys in control group, *p* = 0.29) and in the distribution over the three BART conditions (*p* = 0.48). Moreover, the three BART conditions were similar in boy/girl ratio (68% boys in solo condition, 60.7% boys in the positive risk encouragement condition, and 61.4% boys in the negative risk encouragement condition, *p* = 0.43), distribution of age (*p* = 0.06), RPI (*p =* 0.07*)*, and Raven’s SPM scores (*p* = 0.25). With regard to Raven’s SPM scores, Levene’s test for equality of variances revealed that the variances were unequal. However, this is not a problem with approximately equal group sizes (Bathke 2004). The assumption of homogenous regression lines was met for Raven’s SPM scores as age did not interact with MBID, *F*(4,343) = 1.54, *p* = 0.19, *η*_*p*_^*2*^ = 0.02. Therefore, age was included as a covariate. An ANCOVA showed that MBID was a significant predictor of Raven’s SPM score (*F*(1,353) = 617.08, *p* < 0.001, *η*_*p*_^*2*^ = 0.64) and that age was a significant covariate (*F*(1,353) = 6.42, *p* = 0.01, *η*_*p*_^*2*^ *=* 0.02). A post-hoc *t*-test allowing unequal variances showed that the MBID group (*M* = 28.42, *SD* = 9.03) indeed had a significantly lower Raven’s SPM score than the control group (*M* = 49.25, *SD* = 5.27; *t*(298.11) = 26.79, *p*^*B*^ < 0.001, *d =* 2.82), indicating IQ differences in the expected direction.

### ANCOVA on BART Number of Adjusted Pumps

**Fig. 2:**
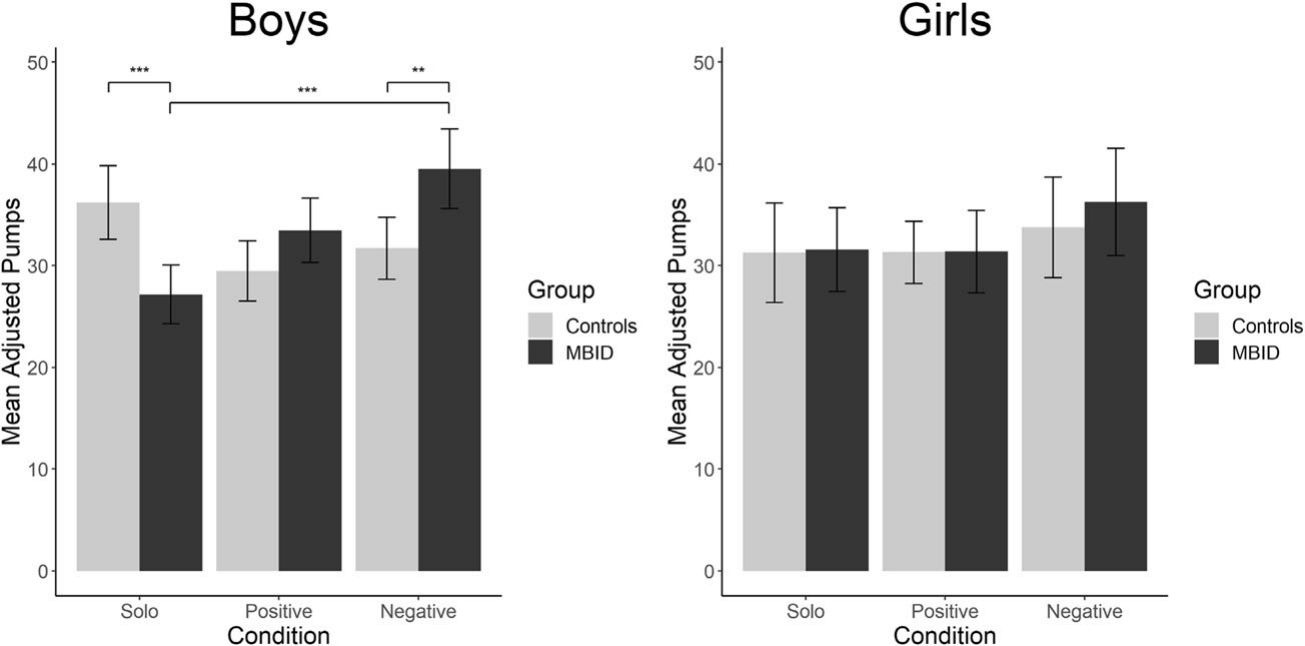
Mean Number of Adjusted Pumps (i.e. Risk Taking) and 95% Confidence Intervals in the MBID and Control Group for Boys and Girls Separately in the Solo, Positive and Negative Risk Encouragement Condition. Note: All significant comparisons are denoted with brackets and stars: ** = *p*^*B*^ < 0.01, *** = *p*^*B*^ < 0.001.

A factorial ANCOVA with age as covariate was performed on BART number of adjusted pumps (see Table 1 for means and *SD*’s and Fig. 2). In line with our first expectation, the ANCOVA yielded a significant main effect of BART condition: *F*(2,343) = 4.98, *p* = 0.007*, η*_*p*_^*2*^ *=* 0*.*03. Post-hoc analyses showed that the adjusted number of pumps was significantly higher in the negative risk encouragement condition as compared to the solo condition (*∆M* = 3.87, *p*^*B*^ = 0.01, *d =* 0.34). However, there was no significant difference in the number of adjusted pumps between the positive risk encouragement condition and the solo condition. Age was not a significant covariate.

**Table 1:** *M* and *SD* on the number of adjusted pumps (i.e. Risk Taking) for the total sample, the MBID group, the control group, and for boys and girls separately in the solo, positive and negative risk encouragement condition

	Total sample			MBID			Control		
	*N*	*M*	*SD*	*N*	*M*	*SD*	*N*	*M*	*SD*
Solo	125	31.42	11.13	70	28.73	10.25	55	34.86	11.34
Positive risk encouragement	117	31.36	9.12	57	32.62	9.60	60	30.17	8.55
Negative risk encouragement	114	35.29	11.53	57	38.16	12.23	57	32.43	10.09
	Total sample boys			MBID boys			Control boys		
	*N*	*M*	*SD*	*N*	*M*	*SD*	*N*	*M*	*SD*
Solo	85	31.41	11.65	45	27.15	9.88	40	36.20	11.73
Positive risk encouragement	71	31.38	9.44	34	33.46	9.41	37	29.46	9.18
Negative risk encouragement	70	35.39	11.11	33	39.54	11.48	37	31.70	9.48
	Total sample girls			MBID girls			Control girls		
	*N*	*M*	*SD*	*N*	*M*	*SD*	*N*	*M*	*SD*
Solo	40	31.46	10.06	25	31.57	10.48	15	31.27	9.68
Positive risk encouragement	46	31.34	8.71	23	31.38	9.96	23	31.31	7.48
Negative risk encouragement	44	35.14	12.29	24	36.27	13.21	20	33.77	11.28

N = Number of participants, M = Mean, SD = Standard Deviation, MBID = Mild to Borderline Intellectual Disability

Contrary to our second expectation, there was no main effect of MBID: *F*(1,343) = 0.50, *p* = 0.48, *η*_*p*_^*2*^ = 0.001. However, in line with our second expectation, the interaction between MBID and BART condition was significant: *F*(2,343) = 5.71, *p* = 0.004, *η*_*p*_^*2*^ *=* 0*.*03. To follow-up this interaction, we performed three post-hoc *t*-tests to compare the MBID and the control group for each BART condition separately. As expected, the MBID group had a significantly higher number of adjusted pumps in the negative risk encouragement condition than the control group, *t*(112) = 2.73, *p*^*B*^ = 0.02, *d* = 0.51. Unexpectedly, in the positive risk encouragement condition this was not the case, *t*(111.89) = 1.46, *p*^*B*^ = 0.147, *d =* 0.27. In the solo condition, the MBID group unexpectedly had a significantly lower number of adjusted pumps than the control group, *t*(123) = −3.17, *p*^*B*^ *=* 0.006, *d* = 0.57.

We explored the main effects and interactions of sex. Of all effects, only the three-way interaction between MBID, BART condition and sex was significant, *F*(2,343) = 4.01, *p* = 0.02, *η*_*p*_^*2*^ *=* 0*.*02. To understand the pattern of interaction, we performed two post-hoc ANCOVA’s for boys and girls separately. In the model for boys, the interaction between MBID and BART condition remained significant, *F*(2,219) = 13.64, *p*^*B*^ < 0.001, *η*_*p*_^*2*^ *=* 0.11. The ANCOVA on girls showed no significant effects. To follow-up this interaction in boys, we first performed three post-hoc ANCOVA’s on the effect of MBID within each BART condition. These showed that in the negative risk encouragement condition, boys with MBID had a significantly higher number of adjusted pumps than boys in the control group, *F*(1,67) = 7.20, *p*^*B*^ = 0.03, *η*_*p*_^*2*^ = 0.10 . There was no such significant difference in the positive risk encouragement condition. However, in the solo condition this pattern was reversed: boys with MBID had a significantly lower number of adjusted pumps than boys from the control group, *F*(1,82) = 15.98, *p*^*B*^ < 0.001, *η*_*p*_^*2*^ = 0.16. Second, we performed six post-hoc ANCOVA’s on the effect of condition (solo vs. positive, solo vs. negative and positive vs. negative) in the MBID and control group separately. These showed that within the group of boys with MBID the number of adjusted pumps in the negative risk encouragement condition was higher than in the solo condition, *F*(1,124) = 22.92, *p*^*B*^ < 0.001, *η*_*p*_^*2*^ = 0.16. The remaining ANCOVA’s did not show significant condition effects. Third, we performed six post-hoc ANCOVA’s on the effect of sex (boys vs. girls) within the each of the BART conditions for the control and MBID group separately. These analyses did not show any significant results.

The exploration of risk taking in the positive risk encouragement condition compared to risk taking in the negative risk encouragement condition showed that in the whole sample the number of adjusted pumps was significantly higher in the negative risk encouragement condition than in the positive risk encouragement condition (*∆M* = 3.93, *p*^*B*^ = 0.01, *d* = 0.38). In all subgroups, there were no significant differences in the number of adjusted pumps between the negative and positive risk encouragement condition.

Explorative analyses within the 144 adolescents with MBID with school file information about DSM-IV classifications showed no significant main effect of comorbid externalizing disorders on risk taking: *F*(1,131) = 0.14, *p* = 0.71, *η*_*p*_^*2*^ *=*0.001, no interaction with sex: *F*(1,131) = 0.03, *p* = 0.87, *η*_*p*_^*2*^ = <0.001, no interaction with BART condition: *F*(2,131) = 1.64, *p* = 0.20, *η*_*p*_^*2*^ *=* 0.02, and no three-way interaction: *F*(2,131) = 2.33, *p* = 0.10, *η*_*p*_^*2*^ *=* 0.03. Additionally, when the main analysis was repeated without 36 MBID adolescents with a comorbid disorder, the pattern of results was similar (*N* = 320, a significant main effect of BART condition: *F*(2,307) = 3.59, *p* = 0.03, *η*_*p*_^*2*^ = 0.02, no main effect of MBID: *F*(1,307) = 0.22, *p* = 0.64, *η*_*p*_^*2*^ = 0.001, a significant MBID by BART condition interaction: *F*(2,307) = 4.67, *p* = 0.01, *η*_*p*_^*2*^ = 0.03, and a significant three-way interaction between MBID, BART condition, and sex: *F*(2,307) = 4.31, *p* = 0.01, *η*_*p*_^*2*^ = 0.03). Moreover, a highly similar pattern of results was found when continuous Raven’s SPM scores were used in the main analysis instead of MBID as categorical variable (see Appendix 2).

### Correlation between RPI and BART

In the whole sample, the correlation between the RPI score and the number of adjusted pumps in the negative risk encouragement condition was significantly negative (*r* = −0.22, *p* = 0.018, see Table 2). This small to medium relation suggests that the more resistant to peer influence adolescents claim to be, the less risks they took in the BART when peers negatively encouraged risk taking. The correlation between RPI score and the number of adjusted pumps in the positive risk encouragement condition was also negative, but not significant. The correlations within the MBID and control group separately were not significant. The follow-up ANCOVA’s in the negative and positive risk encouragement condition separately showed no significant main effects of RPI (negative: *F*(1, 109) = 1.76, *p*^*B*^ = 0.37, *η*_*p*_^*2*^ *=* 0.02; positive: *F*(1,112) = 2.01, *p*^*B*^ = 0.32, *η*_*p*_^*2*^ *=* 0.02) and MBID (negative: *F*(1, 109) = 1.75, *p*^*B*^ = 0.38, *η*_*p*_^*2*^ *=* 0.02; positive: *F*(1,112) = 0.06, *p*^*B*^ > 1, *η*_*p*_^*2*^ = 0.001), no significant interaction between RPI and MBID (negative: *F*(1, 109) = 0.90, *p*^*B*^ = 0.69, *η*_*p*_^*2*^ *=* 0.01; positive: *F*(1,112) = 0.19, *p*^*B*^ > 1, *η*_*p*_^*2*^ *=* 0.002), and age was not a significant covariate (negative: *F*(1, 109) = 0.50, *p*^*B*^ = 0.96, *η*_*p*_^*2*^ *=* 0.01; positive: *F*(1,112) = 0.44, *p*^*B*^ > 1, *η*_*p*_^*2*^ = 0.004). This demonstrates that the relations between the RPI and the BART were not significantly different for the MBID group and the control group. All correlations are shown in Table 2.

**Table 2:** Partial Correlations (*r*) between RPI score and the Number of BART Adjusted Pumps separately for all BART conditions in the Total Sample, the MBID group and the Control Group

	Total sample		MBID		Control	
	*N*	*r*	*N*	*r*	*N*	*r*
Positive risk encouragement	117	−0.16	57	−0.10	60	−0.18
Negative risk encouragement	114	−0.22*	57	−0.23	57	−0.06

* = *p* < 0.05

## Discussion

The current study investigated the effects of MBID, sex, and type of peer encouragement on risk taking in adolescents. To this end, boys and girls with MBID were compared to typically developing boys and girls on an experimental risk-taking task with no, positive or negative risk encouragement by peers. Partly in line with our first hypothesis, negative risk encouragement by peers (e.g., ‘*If you quit, you are a softy’*) was related to higher risk taking compared to solo risk taking, but positive risk encouragement by peers (e.g., *‘Continuing is cool’*) was not. In contrast to our second hypothesis, adolescents with MBID did not take more risks than typically developing adolescents in general, but they did take more risk when peers negatively encouraged risk taking. Third, the exploration of (a) sex differences showed that the abovementioned effects were mainly driven by boys with MBID. Unexpectedly, boys with MBID took less risks without peer influence than typically developing boys. The exploratory comparison of (b) positive and negative risk encouragement showed that negative risk encouragement by peers was related to more risk taking than positive risk encouragement. Fourth, we found that that adolescents who reported more resistance to peer influence took less risks when peers negatively encouraged risk taking.

With respect to our first hypothesis on the effect of peer influence on risk taking, negative risk encouragement by peers was related to more risk taking than no peer influence. This is in line with three lines of evidence showing that social exclusion and peers’ negative interactional styles are related to reputation management, stress causing decreased inhibition, and risk taking (Bjork and Pardini 2015; Blakemore 2018; Brechwald and Prinstein 2011; De Houwer and Tibboel 2010; Ellis et al. 2018; Gunther Moor et al. 2014; Nesdale and Lambert 2008). Adolescents did not demonstrate more risk taking when peers positively encouraged risk taking than without peer influence. Arguably, the potential social exclusion impression is more pronounced in negative as compared to positive risk encouragement (cf. Nesdale and Lambert 2008). The differential effects of negative and positive risk encouragement on risk taking therefore suggest that future studies on the effects of peer influence should focus on negative risk encouragement. Potential working mechanisms can be investigated by using physiological indicators of stress during the peer influence manipulation, or by assessing the need-to-belong as a potential mediator.

With respect to our second hypothesis on the effect of MBID and peer influence on risk taking, we found that although adolescents with MBID did not take more risks than typically developing adolescents in general, they did when peers negatively encouraged risk taking. Moreover, the effect of MBID on susceptibility to peer influence was robust as it was not driven by comorbid disorders in general or moderated by comorbid externalizing disorders specifically. The latter finding matches earlier findings in adolescents with MBID (Bexkens et al. 2018), but is not in line with the fact that decreased cognitive control, as often found in adolescents with MBID (Bexkens et al. 2014), is related to more risk taking in general (e.g., Bjork and Pardini 2015). However, the results specify the general views that adolescents with MBID are highly susceptible to peer influence and have low risk-awareness in peer situations (Dekkers et al. 2017; Greenspan et al. 2011; Khemka et al. 2009), by showing that this is only the case when peers negatively encourage risk taking. As positive risk encouragement by peers was not related to more risk taking in adolescents with MBID, we conclude that the abovementioned potential mechanisms of negative risk encouragement by peers may apply more to adolescents with MBID than to typically developing adolescents. Potentially, an additional decrease in inhibition on top of the already decreased cognitive control in adolescents with MBID (Bexkens et al. 2014), could have made them take even more risks than typically developing adolescents in the same context. Nevertheless, note that the current study was performed in a MBID sample recruited at special vocational schools. It is possible that positive risk encouragement by peers can increase risk taking in adolescents with MBID who show pronounced deviant behavior (Dishion et al. 1999; Vitaro et al. 2000). Therefore, future research in criminal justice system settings is recommended.

With regard to the first part of the third question on potential sex differences, boys with MBID were susceptible to negative risk encouragement by peers, whereas girls with MBID were not. This finding is in line with some research on sex differences in susceptibility to peer influence in typically developing adolescents (De Boer et al. 2017; Sumter et al. 2009; Widman et al. 2016) and with the only study in adolescents with MBID known so far (Dekkers et al. 2017). Moreover, the finding builds on earlier research in which boys with MBID were more susceptible to mixed positive and negative risk-encouraging statements than typically developing boys (Bexkens et al. 2018), by showing that their susceptibility is limited to negative risk encouragement by peers. Thus, the exact effect of peer influence on risk taking seems to depend on specific combinations of adolescent and task characteristics. With regard to task characteristics, our risk-taking task included explicit peer influence of same-sex peers. Potentially, girls with MBID are be more susceptible to implicit peer influences such as indirect bullying (Svahn and Evaldsson 2011) or to opposite sex peer influence related to prostitution-related crime (Kuosmanen and Starke 2015). Nevertheless, many more variations in peer influence situations exist. Therefore, we recommend future research to be aware of the complex interplay between adolescent and task characteristics when designing peer influence paradigms.

Unexpectedly, boys with MBID took less risks without peer influence than typically developing boys. This is not in line with studies demonstrating that adolescents with MBID show higher daily life risk taking than typically developing adolescents (Holland et al. 2002; Kaal 2016; Van Duijvenbode and Van der Nagel 2019). A potential explanation could be that the observed high risk taking in adolescents with MBID often occurs in peer contexts (e.g., Steinberg and Morris 2001). This is also in line with our result that boys with MBID took more risks than typically developing adolescents when peers negatively encouraged risk taking. Thus, a new hypothesis could be that boys with MBID only take more risks than typically developing boys in peer contexts and not when alone. This idea suits the earlier findings of Bexkens et al. (2018) in which boys with and without MBID took the same amount of risks without peers in the solo condition, and even more our unexpected finding of less risk taking without peer influence in boys with MBID. Future research should further elucidate the relation between MBID and risk taking without peer influence in both experimental settings and daily life.

With regard to the second part of the third question, on the comparison of positive and negative peer encouragement, negative risk encouragement by peers led to more risk taking than positive risk encouragement. This is in line with earlier research comparing these two types of risk encouragement (Nesdale and Lambert 2008).

Finally, with regard to our fourth question on the relation between the RPI and the BART, we found that lower self-reported RPI was related to higher risk taking in the BART negative risk encouragement condition. This is in line with earlier research in which adolescents with low RPI took more risks after social exclusion than adolescents with high RPI (Peake et al. 2013). In contrast, a study in typically developing adolescents was not able to detect a correlation between self-reported RPI and risk taking in a version of the BART with neutral peer statements (e.g., ‘*Pump more’*; Cavalca et al. 2013). As the RPI provides an indication of real-life susceptibility to peer influence, this suggests that our addition of negative risk encouragement by peers may have increased the ecological validity of the BART.

Several limitations of the current study may have influenced the results. First, as we used a between-subjects design, we cannot claim variations in risk taking within an adolescent under different types of peer influence. Future studies are encouraged to incorporate a within-subjects design in which adolescents receive at least a solo condition and a risk encouragement condition. This paradigm could be used to derive norm scores for the peer influence effect. Deviation from this norm can then be determined for each individual. With this single-participant approach, those adolescents with MBID most likely to engage in risk-taking behavior as a consequence of peer influence could be identified.

Second, an alternative explanation for the finding that positive risk encouragement by peers was not related to increased risk taking could be that adolescents did not believe this manipulation. Some adolescents in the positive risk encouragement condition indeed showed some signs of disbelief (e.g., *‘You are kidding me, right?’*). However, the same type of peer manipulation with mixed positive and negative risk encouraging statements convincingly produced increased risk taking as compared to no peer influence in Bexkens et al. (2018). Combined with our findings, this may suggest that peers who provide only negative statements or mixed statements may be more credible than peers who only provide positive statements. Unfortunately, our exit-interview was too limited to investigate belief of the peer manipulation (see Appendix 1). Future studies are encouraged to implement a more complete exit-interview and to use more interactive ways of manipulating peer influence such as an online chatroom to increase credibility (see e.g., Weigard et al. 2014).

Third, the age range of adolescents between 12 and 19 years was rather broad. Age differences may have affected the outcomes since previous work suggests a peak in susceptibility to peer influence around age 14 (Berndt 1985; Steinberg and Monahan 2007; Sumter et al. 2009). However, we did not observe any main effects of age or interactions with age on risk taking. Given these null findings for age, and given the fact that age is strongly correlated with pubertal status (Braams et al. 2015), it is also unlikely that pubertal status played a role in our findings. Moreover, pubertal development is not different in adolescents with and without intellectual disability (De Graaf and Maris 2014; Nazli and Chavan 2016). Nevertheless, pubertal status remains a strong predictor of risk taking in adolescence, even above and beyond age (Collado et al. 2014). Future studies are encouraged to study developmental and pubertal trends in susceptibility to peer influence, especially in MBID, by for example incorporating longitudinal designs.

Fourth, we did not study whether adolescents with and without MBID engage in different types of risk encouragement in daily life. As adolescents with MBID are often aggregated in special education classrooms, more deviant peer networks could be formed than in regular schools (Müller 2010). Potentially, this affects the risk encouragements that adolescents with and without MBID provide. Future research should study how often and what types of risk encouragement adolescents provide.

Fifth, the reward component of the BART could have been too abstract for adolescents with MBID. A total of 10 of 59 adolescents with MBID who completed the exit interview indicated that they did not understand that a raffle ticket was received for each 100 points in the game. Future research could include a more concrete reward such as earning actual money for each cashed balloon.

The findings of this study emphasize that awareness of the complexity of susceptibility to peer influence in MBID is crucial for clinical practice. Prevention and intervention programs aimed at reducing risk taking should incorporate individual and contextual factors. The current study was the first to find that especially boys with MBID take more risks when peers belittle or threaten with exclusion. If future studies replicate these findings, interventions could be targeted or adapted to this group and context. An illustration of a suitable intervention could be a decision-making curriculum with hypothetical situations involving negative risk encouragement by peers, which was proven to successfully increase self-protective decision-making and risk perception in adolescents with intellectual disabilities compared to a control training (PEER-DM, Khemka et al. 2016). Based on our results, peer-guided interventions promoting prosocial or healthy behavior via peers (see Stanish and Temple 2012 for an illustration) could focus on decreasing negative peer encouragement on desired behavior in boys with MBID. Our findings suggest that negative peer encouragement may harm the efficacy of the intervention, but this potential effect requires empirical testing. Relatedly, peers could be used as promotors of positive behavior. For example, recent studies show that positive peer feedback on prosocial behavior increases this behavior in typically developing adolescents and adolescents with autism (Choukas-Bradley et al. 2015; Van Hoorn et al. 2017). Potentially, peer feedback could also be used in interventions to decrease risk taking, particularly adolescents who are highly susceptible to peer influence could benefit from this approach.

The current study demonstrated the power of peer influence on risk taking. To return to our question: ‘*When do those “risk-taking adolescents” take risks?*’, we now provided first evidence that boys with MBID are “risk-taking adolescents” when peers belittle or threat with exclusion from the peer group. Although our findings require replication, we stress that more knowledge about specific peer contexts in which at-risk groups show risk taking is essential to decrease future risk taking in adolescents.

## Appendix 1. Exit Interview in the MBID Group

### Comprehension of the BART-Related Reward

59 participants answered the exit question ‘Was it clear for you that you received a raffle ticket for each 100 points in the game?’. 49 respondents answered ‘yes’ and 10 participants answered ‘no’, suggesting that the majority of the MBID group understood the reward system.

### Distinction between Positive and Negative Risk Encouragement

34 participants in the positive or negative risk encouragement conditions answered the exit question ‘Did you like the peers?’. From the 15 respondents in the positive condition, 12 participants answered ‘yes’, 2 ‘sometimes’ and 1 ‘no’. From the 19 respondents in the negative condition, 4 answered ‘yes’, 2 ‘sometimes’, and 13 ‘no’. 31 participants in the positive or negative risk encouragement conditions answered the exit question ‘How kind did you think the peers were?’. From the 18 respondents in the positive condition, 8 participants answered ‘neutral’, 7 ‘kind’ and 3 ‘very kind’. From the 13 respondents in the negative condition, 3 participants answered ‘neutral’, 6 ‘unkind’ and 4 ‘very unkind’. These results suggest that the distinction between positive and negative risk encouragement was clear for the MBID group.

### Faith in the Risk Encouragement by Peers

34 participants in the positive or negative risk encouragement conditions answered the exit questions ‘Did you trust the feedback of the peers?’. 15 respondents answered ‘no’, 17 ‘sometimes’ and only 2 answered ‘yes’. Additionally, in the open question ‘remarks’ two participants answered that they did not listen to the feedback of the peers. This suggests that the MBID group had little faith in the risk encouragement of peers.

## Appendix 2. Intelligence as continuous predictor of risk taking

The main analysis was performed again with Raven’s SPM scores as continuous variable instead of MBID as categorical variable. Total Raven’s SPM scores were converted to percentiles and corrected for age (Pearson Assessment and Information 2006). The pattern of results was highly similar: a significant main effect of BART condition: *F*(2,343) = 8.00, *p* < 0.001, *η*_*p*_^*2*^ = .05, no main effect of Raven’s SPM: *F*(1,343) = 0.16, *p* = 0.69 *η*_*p*_^*2*^ < .001, a significant BART condition by Raven’s SPM interaction: *F*(2,343) = 1.56, *p* = 0.02, *η*_*p*_^*2*^ = .02, and a marginally significant three-way interaction between BART condition, Raven’s SPM and sex: *F*(2,343) = 3.02, *p* = 0.05, *η*_*p*_^*2*^ = .02.

## Footnotes

1 We acknowledge that with the introduction of DSM-5 (American Psychiatric Association 2013), the severity of the intellectual disability is no longer primarily defined by IQ, but by the severity of limitations in adaptive functioning. However, as the DSM-IV was in use when this study was conducted (American Psychiatric Association 2000), the MBID assignment mainly focused on IQ (borderline intellectual functioning: IQ between 70 and 85, or mild intellectual disability: IQ between 50 and 85).

2 The effects of MBID and sex on RPI were not examined as these analyses were already published elsewhere (Dekkers et al. 2017).
